# “I don’t want to make trouble”: Emotional Distress, Disconnection, and Loneliness Among Older People in China

**DOI:** 10.1093/geronb/gbaf068

**Published:** 2025-04-08

**Authors:** Qian Gao, Matthew Prina, Yueqin Huang, Zhaorui Liu, Julia Rozanova, Rosie Mayston

**Affiliations:** School of Public Health, Imperial College London, London, UK; Department of Health Service and Population Research, Institute of Psychiatry, Psychology, and Neuroscience, King’s College London, London, UK; Population Health Sciences Institute, Faculty of Medical Sciences, Newcastle University, Newcastle upon Tyne, UK; Department of Health Service and Population Research, Institute of Psychiatry, Psychology, and Neuroscience, King’s College London, London, UK; Department of Social Psychiatry and Behavioral Medicine, Institute of Mental Health, Peking University, Beijing, China; Department of Social Psychiatry and Behavioral Medicine, Institute of Mental Health, Peking University, Beijing, China; Department of Health Service and Population Research, Institute of Psychiatry, Psychology, and Neuroscience, King’s College London, London, UK; Department of Global Health & Social Medicine & King’s Global Health Institute, Social Science & Public Policy, King’s College London, London, UK; (Social Sciences Section)

**Keywords:** Healthy aging, Qualitative research, Social deficits

## Abstract

**Objectives:**

Loneliness affects more than a quarter of older people. The particular sociodemographic characteristics of Chinese society mean that there are growing numbers of older people, with fewer adults of working age to support them. We explored the experience, meaning, and consequences of loneliness for Chinese older adults, as well as the strategies deployed to counteract unmet social needs.

**Methods:**

This qualitative study was nested within the 10/66 DRG LIFE2YEARS study in China. We conducted in-depth interviews with 49 older people and 13 caregivers. Interviews covered experiences of aging, including social life, expectations of health and social care, and psychosocial support.

**Results:**

We used thematic analysis and developed three main themes: (a) the interconnection of negative emotions, depression, and social deficits; (b) reasons for social deficits—avoiding “making trouble,” social exclusion/isolation, intergenerational strain; and (c) addressing unmet social needs. Older adults experienced loneliness as unexpected pain in later life. They practiced withdrawal behaviors to avoid social embarrassment and perceived risks to health. Older participants described living in communities where they felt increasingly alienated. Whilst some older people described effective strategies for counteracting loneliness, these were only feasible for those who were fit and able.

**Discussion:**

Our findings relating to the shame of loneliness and avoiding burdening others are particularly salient against a backdrop of Confucian ideals. Our work highlights the importance of considering cultural expectations and values in loneliness research and the need to ensure those most at-risk of loneliness are not excluded from future research and intervention development.

Loneliness is a subjective measure of unmet social needs, capturing perceived deficits in either the quality or the quantity of social relationships (Perlman & Peplau, 1981). As well as being a distressing experience in and of itself, loneliness is bad for older people’s health: the effect of being lonely on mortality is comparable to smoking 15 cigarettes a day (Holt-Lunstad et al., 2015). Loneliness is common, affecting one in four older people, and is associated with dementia, heart disease, stroke, and poor psychological health (Hodgson et al., 2020; Rafnsson et al., 2020; Valtorta et al., 2016; Van As et al., 2022). In our synthesis of qualitative studies carried out in lower and middle-income countries (LMICs), we found rejection, overthinking, and pain were common features of loneliness (Akhter-Khan et al., 2024). Across different cultural settings, participants linked loneliness to depression (Adams et al., 2004; Gao et al., 2021). This is consistent with a previous systematic review, which found loneliness/social isolation to be a prominent feature of depression in LMIC settings (Haroz et al., 2017). Similarly, “thinking too much” has previously been identified as a core feature of depression-like illnesses associated with “staying at home,” thereby exacerbating social isolation (Mayston et al., 2020; Patel, 1995).

There is a broad social science literature related to the social needs of older adults. Antonucci’s social convoy model described the supportive web of social relationships, which may ebb and flow but follow an individual across the life course (Antonucci, 2001; Kahn & Antonucci, 1980). The role of culture and the relevance of Western theories outside of the global north, where independence and living alone are often valued positively and coresidence with adult children is not expected, is as yet untested in different cultural settings. More recently, researchers have started to theorize loneliness in older age. Evidence is gradually growing to support the idea that social context should be considered equal to mental and physical health status in determining overall health and well-being. Relationships are the primary mechanism for the distribution of resources to support economic security, health, and functioning in older age, and peer-to-peer and intergenerational interactions are necessary to sustain well-being and sense of self (Bongaarts & Zimmer, 2002). Intergenerational relationships are evolving with societal and environmental change: migration, extended periods of education, and shifting gender roles, leading to unmet expectations of familial support in later life (Du, 2013; Feng et al., 2020; Mayston et al., 2017; Oppong, 2006). We developed the Social Relationship Expectations (SRE), a theoretical framework informed by empirical work from the Global South, which maps the various domains of older people’s relationship expectations (respect, proximity, support, intimacy, fun, and generativity) and hypothesizes that loneliness occurs when these are not met (Akhter-Khan et al., 2023). In our systematic review (Akhter-Khan et al., 2023), each of these six domains was present in over half of the studies included, providing an indication of the utility of this approach. However, as we noted in our conclusions, primary qualitative studies are needed to better understand the role of sociocultural and economic context in shaping relationship expectations.

China’s proportion of over 65 grows rapidly and is projected to reach 26% (366 million) by 2050 (United Nations, 2019). Exacerbated by the one-child policy, China currently faces specific challenges related to a rapidly aging population, echoes of which are seen globally, in countries where fertility has “naturally” declined and populations have aged. This has put increasing pressure on 200 million “4-2-1 families” in China, composed of four older people, one couple, and one child (Zhou et al., 2021) in terms of intergenerational care and economic support. Alongside a rapid increase in the number of older people, there has been a parallel rise in the proportion living with a disability, and at least 26% of older Chinese are now estimated to be disabled (Zheng et al., 2022). There have been recent reforms to pensions and healthcare/insurance, leading to near-universal coverage of health insurance and increased accessibility of healthcare (Meng et al., 2019; Zhong & Jun, 2019). However, our recent analysis of nationally representative data suggested that around 13% of older Chinese people have unmet healthcare needs due to not having enough money, being unwilling to go to hospital,and believing their illness to not be serious (Gao et al., 2022). In 2019/2020, we carried out a qualitative study with older Chinese people living with unmet health and social needs (and their carers) to better understand lived experience and to explore the reasons for unmet needs. In our analyses of unmet healthcare needs (Gao et al., 2024), we revealed that unmet needs for healthcare were experienced through the lens of dislocated relationships with adult children due to geographical distance and willingness/availability to provide emotional and practical support. It was largely impossible for participants to meet their healthcare needs independently due to impenetrable healthcare systems, inhospitable institutions, distance, mistrust in healthcare, and the upfront costs of accessing care (Gao et al., 2024), all of which necessitated the assistance of family members in order to access care. Whilst this analysis provided an explanation for how unmet social needs might contribute to unmet healthcare needs, we wanted to explore Chinese older people’s experiences of unmet social needs, captured in our study through the indication of loneliness, in more depth—both in terms of phenomenological experience, individuals’ understandings of how and why their social needs came to be unmet and the steps they took to address the gap.

## Materials and Methods

### Study Design

This qualitative study was nested within the third wave of 10/66 Dementia Research Group (DRG) LIFE2YEARS work in China. The 10/66 DRG was originally designed to address the deficit in research on dementia and other chronic health problems [capture changing health, particularly cognitive health, and explore determinants] of older adults in rural and urban LMIC sites. The 10/66 DRG study was carried out among older people (65 years old and older) living across 11 geographical catchment areas from eight LMICs, including Cuba, Dominican Republic, Puerto Rico, Venezuela, Peru, Mexico, China, and India (Prina et al., 2017). The longitudinal cohort has three completed waves to date over a period of 12 years (2004–2018). Although this study was embedded within the 10/66 DRG study, this project does not focus on participants with dementia. We used data from survey questions on unmet needs to identify a subset of participants who reported having all five unmet needs for comfort and shelter, food, medical care, clothes and other necessaries of daily life, and travel; and/or reported experiencing unmet social needs (as measured by single-item self-reported loneliness) at the LIFE2YEARS survey, and considerations were taken for sample balance in sex, age distribution, and rural–urban areas (Gao et al., 2024). In-depth interviews were conducted to explore older adults and their family caregivers’ lived experiences, perceptions, and beliefs related to unmet health and social needs, as well as exploring ideas about resources/interventions that might be required to meet healthcare and social needs for aging better.

### Data Collection

This study encompassed in-depth interviews (November 2019–January 2020) at both the rural and urban study sites in two districts of Beijing, China, where the third wave of the 10/66 DRG study (LIFE2YEARS) was conducted (Prina et al., 2017). A narrative approach was selected as this study aimed to encourage interviewees to “tell a story” about their own experiences (Moen, 2006). A series of open-ended questions about aging-related experiences, such as social life (social connection and activities), autonomy, expectations/preferences for health and social care, and psychosocial support, were investigated. All included participants were given voluntary informed consent and completed an in-depth interview alone, except in cases where participants asked to attend interviews together with their family members (*n* = 10). Characteristics of the interviewed participants are shown in Table 1. The interviewer is a young female researcher who has a public health background and local fieldwork experience. All interviewees were encouraged to tell detailed narratives, and research diaries were developed to document the different aspects of the interviewing process, such as the points that the researcher might emphasize, giving little weight to certain aspects of the interview process, etc. (Reid et al., 2018).

**Table 1.** Characteristics of Participants

Characteristics	*N*
*Older adults*	49
Age (range)	65–90
Age group, Mean (*SD*) = 75.0 (6.72)	
65–74	28
75–84	15
85–90	6
Area	
Rural	27
Urban	22
Gender	
Female	29
Male	20
Depression:^a^ ICD-10/DSM-IV/EURO-D	5
Chronic illness^b^	21
Unmet needs	49
5-item unmet needs	49
Unmet social needs—loneliness	6
Living arrangement	
Living alone	13
Living together with family members	27
*Interviewed family caregivers (caregivers’ relation to older people)*	13
Spousal caregiver	5
Daughter	4
Son	2
Daughter-in-law	2

*Notes*: *SD* = standard deviation.

^a^Depression was identified regarding the depressive episode diagnoses of the International Classification of Diseases-10 (ICD-10) criteria, the Diagnostic and Statistical Manual of Mental Disorders-IV (DSM-IV), or the Europe Depression Scale (EURO-D).

^b^Participant with at least one of self-reported doctor-diagnosed chronic illnesses, including high blood pressure, heart trouble or angina, stroke, diabetes, arthritis or rheumatism, stomach or intestine problems, low mood (sad, empty, depressed, or lost interest in most things) and depression.

### Data Management and Analysis

The records of interviews were transcribed verbatim in Mandarin, with a subdataset (interviews for 4 out of 20 households) transcribed and translated into English. A bilingual researcher translated the subdataset and reviewed the whole transcript in Mandarin against the recordings. Data management and coding were done with NVivo 12.0. We applied a thematic approach by reading and rereading the transcripts in order to immerse in the data (Gale et al., 2013) and took an inductive approach. Firstly, we began with conducting open coding to the translated transcripts for a subdataset independently by two researchers. Then, two coders met to generate an initial codebook by comparing and grouping the separate coding. After that, the primary coder applied the initial codebook to the whole transcripts in Mandarin, allowing for emerging codes. Meanwhile, an analytical framework was generated by grouping the initial and emerging codes. The primary coder developed a set of themes inductively by reviewing the summaries of coding. All transcripts and themes were reread to ascertain the credibility of themes with original data. Themes and selected quotes were then translated into English and reviewed by the research team for coherence.

## Results

Overall, we interviewed 49 older people and 13 caregivers from 40 households. The sample of older adults consisted of both men and women, with an age range from 65 to 90 years (Table 1). The findings comprised three main themes and subthemes: (A) the interconnection of negative emotions, depression, and social deficits in older age, (B) reasons for social deficits, with subthemes including avoiding “making trouble”; social exclusion/isolation; intergenerational strain, and (C) addressing unmet social needs (Table 2).

**Table 2.** Themes, Subthemes, and Quotations

Theme	Example quotation
(A) The interconnection of negative emotions, depression, and social deficits in older age	Quotation 1: *Sometimes I’m in a bad mood, I feel unhappy, it seems to have developed a little bit, developed a little to depression.” (Rural, ID60, Female, 68 years old, living with a spouse)*
	Quotation 2: *“She must feel a little sad those days. They [old friends] gradually passed away, one after another. Then, the last old lady (who passed away) was younger than her. After she [the old lady] left, she rarely goes out since then. She doesn’t like to go out now because her body is no longer well. You ask, where is she going to visit? Those she is familiar with have passed away. She is unfamiliar with the others (around). Now, she can only stay at home. I think she is anxious about getting old. But I am not sure because she didn’t check in the hospital. If you ask her about this, she will deny it, and she does not admit it [anxiety], but I feel that if a person gets old, her temper is bound to change.” (Urban, ID5-Caregiver(daughter), Female, 65 years old, older participant lives with her daughter’s family)*
(B) Reasons for social deficits	
avoiding “making trouble”	Quotation 3: *“(After having a stroke) I looked at others and thought in my heart, I can do none of these activities, I can’t walk, I just watched them ‘Bengdada’ [having fun] outside, what’s in my heart? I always feel sad, I feel too sad, I can’t do anything.” (Rural, ID37, Female, 82 years old, living with daughter’s family)*
	Quotation 4: *“I can’t walk now. I rarely attend any activities. My children sometimes invite me to go out. They drive to take me out. I have to bring a wheelchair. Sometimes I don’t want to go with them, because I’m quite old, and I always say that I’m making trouble for them. If you can walk, it’s much easier, but now you can’t walk, you always trouble young people. That’s it.” (Urban, ID7, Female, 81 years old, living alone and widowed)*
	Quotation 5: *“That’s a huge change with getting old. Not like before, you’re healthy. Now, all these friends are old, and their family has their affairs. What to do if they feel inconvenient when you visit [friends]? You also need to think more of others. You’re free, but others may be busy. They have children and grandchildren and have things to be busy with, so we rarely contact each other (old friends). We have to consider more for them, so I don’t have any social activities now.” (Urban, ID17, Male, 70 years old, living with a spouse)*
social exclusion/isolation	Quotation 6: *“I know that side (of the village), they have organised a dancing activity. But, you’re old, you can’t follow the dance, they wouldn’t like you old guys to join them. They’re younger and look down on you. I didn’t attend it. They danced several years ago. We, older people, are willing to attend it, but they wouldn’t be willing to let you join. Older people are slower than others when dancing. Don’t make trouble for others.” (Rural, ID46, Female, 72 years old, living with son’s family and widowed)*
intergenerational strain	Quotation 7: *“I think we should have an elderly care home nearby, like a ‘nursery’ for older people in the community. It would be great if they could organise some (social) activities there. I think we should have a place where older people can stay together (to socialise). Otherwise, look outside. It’s too cold to stay outside. We should have a place for older people to stay and play together.” (Urban, ID23-Caregiver (daughter), Female, 58 years old, older participant lives with her daughter’s family)*
(C) Addressing unmet social needs	Quotation 8: *“The community organises it, and then we are on duty, (we are) volunteers, and participate in all the community activities. Otherwise, you have nothing to do at home. (Being a volunteer in the community) is helpful for socialising. We chat with each other and walk around together. We can chat together when we’re on duty. Now I feel quite satisfied. Sometimes, the community will organise our volunteer work members to travel once or twice a year.” (Urban, ID31, Female, 72 years old, living with a spouse)*

### (A) The Interconnection of Negative Emotions, Depression, and Social Deficits in Older Age

Loneliness was a slippery and unpleasant experience for older people, who variously felt upset, sad, bored with no effective way to address this. Some participants described a blurring of experiences they characterized as loneliness with feelings they associated with depression: most commonly, participants felt depression could take hold if they were unable to manage their feelings of loneliness effectively (Table 2, Quotation 1).

Older people and their caregivers described the interconnected, circular relationships between loneliness, distress, and later life. Older people’s emotional unrest was perceived as a direct consequence of life changes linked to aging. Some older participants were experiencing profound distress as a result of perceived losses of care, activity, meaningful social interactions. An older woman who lived with her spouse described her deep sense of helplessness, explaining her distress as being due to a lack of care, which left her feeling isolated on an inevitable pathway to illness and eventual death:

*(We live alone) Sometimes the community staff come to visit us, but it’s just once in a while. Now I feel helpless, just like that. If I’m ill and get hospitalised, and then I’ll pass away, life feels like this, very, how to say it? I think we [the old couple] will follow these steps. We feel sad, we’ve rarely felt like this before. He [husband] cries more than before. I think (we) might be more vulnerable as we get older. (Urban, ID3, Female, 74 years old, living with a spouse)*

Whereas others emphasized how their loneliness meant that there was no outlet for their feelings. One woman expressed despair at her situation—that she was imprisoned by old age and dependence:

*[I] just manage to live, uh, I’m still alive, not dead yet. I’m willing to die and then I won’t burden others anymore. I have nothing to do. Sometimes, I feel upset. (When) you are upset; no one cares (about your emotions). Uh, you’re saying, whom I can speak to? I can only bear it myself. (Urban, ID22, Female, 85 years old, living with daughter’s family and widowed)*

Others accepted loneliness as an unwelcome but unremarkable characteristic of slowing down in later life, which was often unspoken

*I used to do a lot of work when I was younger. Who thought about loneliness when we were younger? We were always busy at that time. Now we are free, we feel a little bit lonely. It’s ok, it’s normal. I don’t know what he feels, he [husband] must feel lonely too, but he never says. (Rural, ID34, Female, 71 years old, living with a spouse)*

Some blamed themselves for their loneliness: for not keeping themselves sufficiently occupied, the consequences of which they therefore needed to manage alone:

*(living alone) I feel “that”, (I feel) lonely. It is like that; sometimes life is not well arranged, too often I have too much free time, and I feel a little lonely. My children are very nice, but sometimes, I don’t know why I still seem lonely, like “that feeling” (it’s hard to describe). I don’t know why I have the feeling. But I try to manage it myself. (Rural, ID60, Female, 68 years old, living with a spouse)*

One man described how he had become more introverted with age, losing interest in social activities, which he also connected to the loss of his wife:

*(My) personality changed gradually. I used to be quite outgoing. Now, I am willing to find a quiet place, stay there and listen to music. It may be caused by being old and also the changes in my family (his wife had cancer and passed away). All these things may be connected. Compared with the past, when I was young, I can say that when my wife was not ill, for example, if there was any activity, I would like to take a look. But now, I avoid those activities. My mentality has changed. It may be linked with being older or the changes in my family life. All may have a connection. (Urban, ID19, Male, 72 years old, living alone and widowed)*

Caregivers generally recognized their older relatives’ discontent, and were saddened by it, naming it as depression or anxiety (which was often repudiated by older people), and perceiving distress to be a natural consequence of the inevitable losses older people experienced and which they associated with aging (bodily function, friends who have died, unfamiliar neighbors) (Table 2, Quotation 2).

### (B) Reasons for Social Deficits

#### Avoiding “making trouble”

Older people described avoiding “making trouble”—causing inconvenience to themselves and others—at all costs. In practice, this meant that older people frequently secluded themselves from social situations where there might be an expectation or need for others, often younger people, to adapt or change their behavior. For example, problems with mobility, which prevented many older participants from exercising or socializing independently were frustrating and upsetting (Table 2, Quotation 3). Older people reported turning down invitations for social events in order to avoid “making trouble” for others (Table 2, Quotation 4).

Older people kept themselves away from situations they perceived to confer a risk of getting sick in order to avoid causing trouble for themselves and potentially others down the line. This meant that outdoor activities during cold weather, for example, were commonly precluded:

*When the weather is warm, I drive my electronic car. But for these cold days, I hide at home. Otherwise, If I catch a cold, I make trouble [for self and family] again. If I need to visit somewhere alone, I always try to avoid it. I don’t want to make trouble. You see, I don’t go out on cold days. She [wife] also doesn’t go out in winter. Don’t make trouble. What to do if you catch a cold? I think it’s safer to stay indoors than outdoors, right? (Rural, ID33, Male, 72 years old, living with a spouse)*

Interviewees directly linked the social disruption caused by participating in social activities as a person living with an age-related disability with experiencing a sense of shame and diminished respect from others:

*Now it is cold outside, and he doesn’t like to go out. He doesn’t want to use the crutch. Our neighbours said, “Use a crutch; it is helpful.” He said (if using it) I will lose face [shame], I don’t use a crutch. (Rural, ID34, Female, 71 years old, living with a spouse)*

Similarly, older people who lacked close relationships with their descendants took care not to burden their friends who were occupied with children and grandchildren with visits, which might be disruptive or cause embarrassment (Table 2, Quotation 5).

#### Isolation and exclusion

Changes in older peoples’ circumstances as well as changes to their communities over time reduced opportunities to socialize. For example, one older woman described how a combination of different types of losses (youth, mobility, friends, and other older people in the neighborhood) led to her feeling isolated and trapped in her community despite the fact that she lived with her daughter’s family:

*I’m over 80 years old. (As I get old*), the*re is a change, I can’t get out now, and there is no one [friend] around. The neighbours are all young people. At that time, all people living around me were getting along well with me. All these people are gone [passed away], and I can’t go out now. I’m old too. They were about the same age as me when they died. They left first, and I’m the only one left. (Urban, ID4, Female, 90 years old, living with* daughter’s family and widowed)

Others described the natural loss of community and sense of belonging associated with work when they retired:

*After you retired, just like this, when you retired to go back home, your social connection changed then, you don’t have ties to your company anymore, right? Your socialising has reduced a little bit. That’s to say that to say that (I) have lost contact with old colleagues. (Urban, ID7, Female, 81 years old, living alone and widowed)*

Caring for a spouse with little or no support from others restricted freedom and appetite to socialize. One older female participant described how taking care of her dependent husband alone tied her to her home, leaving her feeling fatigued and isolated:

*Most time, I don’t visit others. I am too tired to go out now. My husband always needs someone to take care of him. Now, I can’t go anywhere. I want to travel, but I must take care of my husband. I can’t go anywhere. He can’t walk, even for one step. It’s unrealistic for me to go out. But I can’t change it now. It’s been more than three years. No one else can help with this. (Rural, ID53, Female, 70 years old, living with a spouse)*

In both urban and rural communities, older people described lacking physical space for social activities, forcing many to remain isolated at home:

*The facilities here are weak. There’s no place, how can we participate in any social activities? There is no activity in the community. She rarely travels far away from home. It’s been several years, just staying at home. She doesn’t have social activities opportunities for locally. (Rural, ID51-Caregiver (husband), Male, 66 years old, living with a spouse)*

Many felt excluded from community events, uninvited and unwelcomed at social activities that were perceived to be centered on younger people and therefore seen to be unsuitable for older community members (Table 2, Quotation 6).

#### Intergenerational strain

If not coresident, the children of our older participants were mostly living close by. Nonetheless, older people described feeling disconnected from their children, regardless of whether they lived in the same household.

*No, what family activities do we have? Every time they [children] come back home, they just walk around and stay for a little while, then it’s time to go back to their own home. (Rural, ID33, Male, 72 years old, living with a spouse)*

Older people explained their experience in terms of their children being too busy due to work and lacking time to take care of and engage with them:

*You would like to talk with them [children], but they don’t have time to chat with us. They are always busy with their jobs, but they often call us. They don’t come every week. If they’re free, they come to sit for a while. They don’t have time. He (son) is busy. If he doesn’t come to visit us, we can understand. (Urban, ID8, Female, 78 years old, living with a spouse)*

Family tensions led to some older people acknowledging that their relationships with their children were dysfunctional; reluctantly, they let go of any expectations of support, relying instead upon their own self-sufficiency. An older man commented on the importance of not overburdening his sons with unnecessary demands in order to maintain equable relationships that would hold fast against future crises:

*(When older) If you always need children to take care of you, he won’t have that time, now we [the older adult and his sons] don’t have conflicts. I need to pay attention to maintaining this relationship. This is what you must do when you are old, or if you can’t move, no one can help you. (Rural, ID45, Male, 68 years old, living with a spouse)*

Participants alluded to the wider societal context, which contributed to intergenerational strain, including China’s one-child policy and the inequities in government support between older urban and rural residents:

*You can’t rely on your children, and they need to work. It’s impossible. If children take care of you, they have too many burdens. The issue is that one young couple needs to take care of four older adults. It must be very tough. Workers have pensions, but farmers only have a subsidy. Our subsidy can only afford basic daily life. If you need to take medications, you have to rely on your children. (Rural, ID55, Male, 67 years old, living with a spouse)*

Some older people and caregivers suggested that provision of residential care facilities and day centers might help to plug the gap, providing opportunities for older people to enjoy socializing together, taking the pressure off younger generations to provide caregiving and interaction (Table 2, Quotation 7). One older woman highlighted that those without children living nearby were most in need of these kinds of facilities:

*Older people, widowed older people around here, and those living alone are all very depressed. Like me- my children don’t live nearby, and no one can take care of me. If there is a place where older people can stay and chat together, you’ll feel you have some support in older age. (Urban, ID3, Female, 74 years old, living with a spouse)*

### (C) Addressing Unmet Social Needs

Older people described the various activities that they practiced in order to give them a sense of contentment, purpose, and belonging. Most of these were social in nature, involving face-to-face interactions with others, and took place outside of the home:

*I attend some social activities in the community, so I feel happier than before. You can’t always stay at home. I also volunteer to provide (traditional) medical treatment for older people here (nearby). I learn new things on the mobile phone. We have an old neighbour who organised a law class for older people. I always participate. (Urban, ID3, Female, 74 years old, living with a spouse)*

Opportunities to contribute to their local community in the form of volunteering were welcomed as a source of meaningful activities (outside the home) and social interaction, which facilitated additional possibilities for travel outside of the neighborhood (Table 2, Quotation 8).

For some, a continuation of working habits from younger years kept them occupied in later life, helping to keep them in a good mood:

*Sometimes, I repaired bicycles for others, repair tyres, and I’ve learned this since I was young. This helps to improve mood. I just want to do some work, find something to do. I don’t have too many demands, just hope to have something to do in the daytime. I’ll hang out when I’m free. I don’t have anything to worry about. (Rural, ID32, Male, 68 years old, living with a spouse)*

The value of social interaction with younger people was noted in helping to maintain an ability to engage with others in a fast-changing society. Opportunities for social learning with peers were also valued for the joy they brought older people. Others highlighted the importance of learning new skills to keep pace with new technologies:

*(As we age old), How could it not change? People now use that mobile phone, but we don’t know how to use it. I just learned how to make a WeChat payment this year. Ah, they said: ‘You’re Niu [cool], you can use online payment’. I said: ‘It’s inconvenient if you don’t know (how to use) this. I need to learn it. I already know how to use it.’ I plan to learn something else this winter. (Rural, ID44, Female, 70 years old, living with a spouse)*

Some older people commented that they would like to take part in social activities if they were available but that this would need to be supported and coordinated by other community members:

*If the street (committee) can organise some activities, such as older people’s college or other activities. He (husband) would be happy to participate if someone can organise travel programmes for older people. But we need someone to organise it. (Urban, ID13-Caregiver (wife), Female, 70 years old, living with a spouse)*

## Discussion

Older people experienced a visceral shock at the isolation, loneliness, and sadness they experienced as a result of days that felt empty of meaningful social interaction and compassion from others. Many felt helpless to address these problems, particularly in light of lost connections (due to death, people moving out of neighborhoods), lost mobility, lost independence, and the social and health risks they perceived of venturing outside of their homes. Older people described a vicious cycle of withdrawal behaviors that they practiced to avoid making trouble, which exacerbated their isolation and loneliness. At the same time, they described living in communities where there were reduced opportunities for social interaction and where they felt excluded from social events and activities. Older participants highlighted the alienation they often felt from their children and suggested that this was due to demographic and societal change and increased pressures on younger generations in terms of work and caregiving responsibilities.

Loneliness theories often start from a cognitive, psychological standpoint, focusing on the individual and their expectations of social relationships, whilst taking into account varying degrees of the broader social, cultural, and economic context in which these relationships do/do not occur. Our study suggests that it may be necessary to foreground the cultural theories that shape social interaction and how these interact with sociodemographic and economic contexts. It is this dynamic process that may be the key driving force behind loneliness and depression, particularly among older people in societies where there has been rapid social and demographic change and where there is often a disconnect between the sociocultural norms and theories older people were socialized into and the world in which they are growing old.

Consistent with the literature, we found that older participants perceived loneliness to be linked to depression (Adams et al., 2004; Akhter-Khan et al., 2024; Barg et al. 2006; Lee et al., 2021; McHugh Power et al., 2020). Whilst a slowing down of social interactions and an accompanying lack of interest in pursuing social activities was expected in older age, this rarely meant that this was experienced without distress about what was being lost. For many, loneliness formed the early part of the continuum of depression, which, without intervention and effort, could easily become more serious and ingrained (Barg et al. 2006). Consistent with other qualitative work carried out in China, in our interviews, by being committed to “not making trouble,” older people endorsed and embodied Confucian values of harmony and maintaining etiquette in their interpersonal relationships of not bothering others when they have problems (Zeng et al., 2014). As illustrated by one of our participants, ideally, “not making trouble” would be aligned with filial piety and the expectation that adult children provide holistic support to their parents. However, the twenty-first-century reality is that older people’s expectation of intergenerational reciprocity (in China, as well as elsewhere) is frequently not met (Du, 2013). “Not making trouble” therefore amounted to social withdrawal, designed to maintain harmony but resulting in older people feeling outside of the normative system of social exchange (Barg et al. 2006). For some, this sensation was akin to Durkheim’s (1951) concept of *anomie* (Durkheim, 1951): disengaged from society, older people expressed a passive desire for death as an end to their distress. In this sense, our findings suggest that this cultural disjuncture, driven by economic and societal change, can be a driver of both loneliness and depression among older generations. Elsewhere, among people living with HIV, we have noted the conceptual overlap between depression and internalized stigma (Rosie Mayston et al., 2023). Here, there is a sense that because older people feel that their aging bodies disrupt Chinese cultural values of interpersonal harmony, they consequently perceive their social identities to be “spoilt” by older age (Erving Goffman, 2009). These beliefs drive behaviors, which contribute to social isolation, loneliness, and depression.

In addition to changes to close family dynamics, changes to neighborhoods and communities contributed to older people’s sense of alienation at a societal level (Wong et al., 2017). The moving-in of young people to neighborhoods once populated by familiar peers and a sense of being excluded from community events and activities contributed to a sense of a growing distance between older people and the rest of society. Loss of the social self was exacerbated by physical health problems, in particular, lost mobility and sensory impairment, which made attending social occasions practically more difficult, as well as being associated with “trouble” causing others’ embarrassment and resulting in shame. As was suggested by findings from another study in China (Wong et al., 2017), older people’s sense of not being well protected by society made them viscerally aware of their inherent vulnerability, resulting in anxiety and hypervigilance of risks relating to being outside their house in the outside world. Taken together, shifts in close family dynamics and community relationships contributed to an overall sense of a disintegrated social convoy, where older people did not experience the level of support and belonging that they expected (Antonucci, 2001).

The activities and practices older people described as helping them to feel better—more socially connected, purposeful, satisfied, and happier, were aligned with some of those included in the SRE framework (Akhter-Khan et al., 2023). From our study, it is unclear to what extent these peer-to-peer relationships and self-driven activities were sufficient to take the place of involved and compassionate relationships with children that are so important in Chinese culture, which has historically been the primary mechanism for support in older age. The activities described were practiced by a subset of participants who tended to be younger and fitter and therefore more independent. For those who were more frail, needs for support and concerns about causing trouble meant that the self-driven activities that others found so helpful were all but impossible. The coping strategies identified by our older participants are a good fit with those described in the SRE and previous qualitative evidence from other cultures (Mansfield et al., 2021). However, our findings suggest that there are likely to be culturally determined hierarchical relationships between domains, with some being of greater importance in particular contexts, not just in terms of shaping loneliness but also influencing other key drivers of well-being, such as depressive symptoms.

A potential limitation is the purposive sampling in this study, as we recruited a group who has reported all five types of unmet needs in socializing, comfort and shelter, food, medical care, travel, clothes, and other necessaries of daily life, and having self-reported loneliness at a single time point in the 10/66 DRG LIFE2YEARS survey. This survey captured the unmet social needs circumstances two years before our qualitative interview, although we observed limited changes in older people’s circumstances since the LIFE2YEARS survey. It is potential that we captured a relatively poor group with experiences of being left behind that may be particularly severe. The sampling may shape the understanding of unmet social needs. Therefore, the findings are less likely to be generalized to the entire older population in China. Cases with unmet needs were screened based on categorization in questionnaires: there are two potential problems with this. Firstly, they were administered in the past, and the perpetuation of the same conditions was not confirmed. Secondly, admitting unmet needs in a culture where older people’s expectations in later life are shaped by Confucian ideals is potentially stigmatizing. For example, our previous research found a distinctively lower prevalence of loneliness in China than in other study sites in Latin America and India (Gao et al., 2021). However, similar associations with social and health factors in China and other study countries suggest this is an artifact of underreporting. Another limitation was that our sample framework was from two catchments in the capital city, which may be less representative of residents in other cities. Particularly, community characteristics may have shaped our findings, which are not able to be applied elsewhere. Moreover, we acknowledged that the positionality of a young female researcher, who was educated in China and the UK, may affect the way of interview and interpretation (Reid et al., 2018).

## Conclusion

The phenomenon of self-limiting behaviors to avoid social embarrassment and risks to health combined with communities in which older people feel increasingly alienated has left some older people with unmet social needs: feeling isolated and helpless (Mansfield et al., 2021). Whilst some older people described helpful strategies that supported them to find purpose and new connections, older people who were dependent or disabled were excluded from these possibilities. Our qualitative work thereby provides empirical evidence of mechanisms for associations between disability, dependence, and loneliness, which have been found in China as well as elsewhere in quantitative studies (Gao et al., 2021; Shankar et al., 2017) but lack detailed qualitative exploration (Mansfield et al., 2021). Qualitative research from other cultures described similar expressions of loneliness in older adults, including stigma and wishing to avoid burdening family and others, including a perceived responsibility to hide and control feelings of loneliness (Barg et al., 2006; Drageset et al., 2015; Muir & McGrath, 2018). However, this phenomenon may be particularly salient among older Chinese, given the connection between stigma and the cultural phenomenon of “face” and maintaining “moral” status in domestic and community life, along with Confucian values of harmony, which can explain their cultural reluctance to cause trouble for others (Yang & Kleinman, 2008), despite the pain caused by living with unmet social needs. Our work thereby highlights the importance of considering the power of cultural expectations and values in shaping the meaning and consequences of loneliness.

Given the public health importance of loneliness and the additional barriers to self-help for unmet social needs, it is important that interventions targeting loneliness in older adults do not leave behind the most vulnerable: those who live alone and/or with disabilities and who may be housebound. Whilst China’s sociodemographic and cultural landscape has created a particular set of challenges for twenty-first-century families, these have familiar echoes of dilemmas around the world. Health and social reforms have not provided a replacement for family support (Carbonell et al., 2020; Whiteford, 2003). It remains to be seen whether government intervention for rapid upscaling of long-term care and daycare facilities (Feng et al., 2020) will satisfactorily address older people’s social needs in addition to providing instrumental care. In addition to research to address unmet social needs for older people, it may be necessary to consider intergenerational research with families and communities to raise awareness of the social pain isolated older people experience, the health risks associated with this, and to generate ideas for what can be done to improve cohesion between generations, both within families and wider communities.

## Data Availability

Data supporting the findings of this manuscript are available from the corresponding author, Q. Gao, on request.
